# Axonal degeneration in multiple sclerosis: can we predict and prevent permanent disability?

**DOI:** 10.1186/s40478-014-0097-7

**Published:** 2014-08-27

**Authors:** Jae Young Lee, Kasra Taghian, Steven Petratos

**Affiliations:** Department of Medicine, Central Clinical School, Monash University, Prahran, Victoria 3004 Australia; Melbourne Medical School, Faculty of Medicine, Dentistry and Health Sciences, University of Melbourne, Parkville, Victoria 3010 Australia

**Keywords:** Axonal degeneration, Multiple sclerosis, Biomarkers, NF-L, N-Acetyl aspartate (NAA)/Creatine ratio, Microglia, Sodium channels, Collapsin response mediator protein 2, Calpain

## Abstract

Axonal degeneration is a major determinant of permanent neurological impairment during multiple sclerosis (MS). Due to the variable course of clinical disease and the heterogeneity of MS lesions, the mechanisms governing axonal degeneration may differ between disease stages. While the etiology of MS remains elusive, there now exist potential prognostic biomarkers that can predict the conversion to clinically definite MS. Specialized imaging techniques identifying axonal injury and drop-out are becoming established in clinical practice as a predictive measure of MS progression, such as optical coherence tomography (OCT) or diffusion tensor imaging (DTI). However, these imaging techniques are still being debated as predictive biomarkers since controversy surrounds their lesion-specific association with expanded disability status scale (EDSS). A more promising diagnostic measure of axonal degeneration has been argued for the detection of reduced N-acetyl aspartate (NAA) and Creatine ratios via magnetic resonance spectroscopic (MRS) imaging, but again fail with its specificity for predicting actual axonal degeneration. Greater accuracy of predictive biomarkers is therefore warranted and may include CSF neurofilament light chain (NF-L) and neurofilament heavy chain (NF-H) levels, for progressive MS. Furthermore, defining the molecular mechanisms that occur during the neurodegenerative changes in the various subgroups of MS may in fact prove vital for the future development of efficacious neuroprotective therapies. The clinical translation of a combined Na^+^ and Ca^2+^ channel blocker may lead to the establishment of a *bona fide* neuroprotective agent for the treatment of progressive MS. However, more specific therapeutic targets to limit axonal damage in MS need investigation and may include such integral axonal proteins such as the collapsin response mediator protein-2 (CRMP-2), a molecule which upon post-translational modification may propagate axonal degeneration in MS. In this review, we discuss the current clinical determinants of axonal damage in MS and consider the cellular and molecular mechanisms that may initiate these neurodegenerative changes. In particular we highlight the therapeutic candidates that may formulate novel therapeutic strategies to limit axonal degeneration and EDSS during progressive MS.

## Introduction

Destructive, inflammatory demyelinating multiple sclerosis (MS) lesions can occur throughout the central nervous system (CNS) with preferential anatomical patterns forming. Clinical symptoms in an MS patient may manifest as a range of neurological deficits, including paresthesia, dysesthesia, weakness, or visual disturbances such as blurring or greying of vision and black spots in the visual field (scotoma, a consequence of optic neuritis). There have been several risk factors postulated for the development of MS, namely genetic, inadequate exposure to Vitamin D, smoking, Epstein Barr virus infection early in life and geographical in relation to latitude gradient [1]. In approximately 90% of cases, the disease manifests with an initial primary phase characterized by a relapsing-remitting presentation (RRMS) where the patient experiences alternating episodes of neurological impairment, followed by recovery [2]. The secondary phase involves the transformation of a relapsing-remitting presentation into a secondary progressive MS (SPMS), which involves a persistent neurological decline [2]. In contrast, 10% of patients undergo primary progressive MS (PPMS) where the course of the disease adopts a steady decline in neurological function without any periods of recovery [2]. The prognosis of MS can also vary from complete and lasting remission to eventual paralysis, loss of bowel and bladder control and blindness [3], and even death in the case of the most aggressive form of the disease, acute rapid progressive MS [3].

Therefore, MS is considered a heterogeneous condition where disease features may vary from one patient to another. Despite the different histopathologically characterized lesions of MS, they share common hallmarks that include areas of focal demyelination with inflammatory infiltrating immune cells, along with axonal injury. Contrary to the original focus of research on the autoimmune mechanisms that are operative in MS, there is now clear evidence that axonal damage/loss is the major determinant of profound neurological deficit in MS sufferers. In light of the variable course of the disease and different prognostic outcomes we discuss the possibility that in some subgroups of MS, molecular mechanisms may initiate axonal degeneration as a primary event, preceding inflammatory destruction of myelin, leading to variable capacity for repair and thus variable patient presentation.

This review focuses primarily on why MS may progress to permanent disability. The discussion covers clinical, pathological, cellular and molecular mechanisms, which govern axonal pathology in progressive MS, the etiology of permanent neurological disability. The recent advances in biomarkers and possible molecular mechanisms driving axonal dysfunction through ion channel and axonal transport defects will be highlighted with an emphasis on therapeutic targeting in an attempt to halt axonal pathology and hence progression of the disease.

### Axonal indicators in the neurology clinic

Currently there is no clinical, laboratory, imaging or pathological sign of MS that is pathognomonic for the disease. At present, the diagnosis of MS is based on the two McDonald criteria [3]. The first being that there should be evidence of at least two demyelinating lesions in the CNS that are separated both spatially and temporally. The second criterion is that all other alternative diagnoses are ruled out by clinical investigation. Therefore, the diagnosis of MS essentially remains one of exclusion from the clinical evidence provided and so the need for diagnostic biomarkers is warranted in particular to personalize therapeutic regimes.

Defining axonal damage during MS, a prediction of progression, has proven somewhat problematic since conventional magnetic resonance imaging (MRI) does not provide clinicians with an accurate interpretation of the underlying pathology. Despite numerous biomarkers of axonal damage being recently reported to be superior in their diagnostic and eventual prognostic capacities for MS progression, limitations still exist for their utility in isolation.

#### Current imaging techniques

Further revision of the McDonald criteria in 2010 has led to a more simplified version of MS diagnosis which now include those patients that present with CIS, either as monofocal or multifocal demyelinating lesions, with involvement of the optic nerve, brainstem and cerebellum, spinal cord and cerebral deep white matter tracts [4]. Such slow expanding lesions on progression, can be absent by T1-weighted MRI inspection following gadolinium (Gd) enhancement [2].

Current MRI technologies are elucidating the substantial involvement of axonal degeneration with increasing disability parameters [5], previously difficult to define. High resolution diffusion tensor imaging (DTI) has been used in a rat model of experimental autoimmune encephalomyelitis (EAE), generating data which support the contention that significant axonal damage and loss can occur at some distance from the primary inflammatory lesion, strongly correlating with disability [6]. Alterations in DTI measurements are also well documented in MS patients [7]. Two main parameters that are disturbed in MS patients are mean diffusivity (a quantitative metric of water diffusion) and fractional anisotropy (prevalence of diffusivity along one direction) [8]. Increase in mean diffusivity often reflects edema, axonal and myelin loss [9] whereas reduction in fractional anisotropy indicates demyelination in MS [10]. Increased mean diffusivity and decreased fractional anisotropy were detected in NAWM of MS patients [11]. These changes were more profound in SPMS patients compared with CIS, RRMS and benign MS [12]. Moreover, these diffusion abnormalities were also found in grey matter of MS patients where axonal or neurodegeneration are prominent, shown to be greater in SPMS compared with other MS phenotypes [12]. Attempts have been made to correlate diffusion alteration to EDSS, however, the results of these studies remain controversial [13-19]. Since EDSS is based on motor system criteria, it can be suggested that motor-system specific DTI measurement would have a high correlation with EDSS. In fact, region-specific DTI measurements in MS patients have indicated a greater correlation of DTI changes in motor tracts with EDSS [20]. Importantly, it has been recently proposed that a reduction in axial diffusivity measurements of DTI correlate to extensive neuroinflammatory-mediated axonal damage within the optic nerve following acute optic neuritis, often a primary indicator of MS [21]. Moreover, these investigators demonstrated that protracted reduction in axial diffusivity measurements correlated with pronounced retinal nerve fiber layer (RNFL) thinning and multifocal visual evoked potential (mfVEP) amplitude loss at 12 months. Therefore, the argument for the use of axial diffusivity measures as a predictor of poorer visual outcomes in patients is justified and may in fact be an excellent biomarker for neuroprotective therapies in MS that limit axonal degeneration.

Another non-conventional MRI approach utilized to study axonal degeneration is the magnetization transfer ratio, which has been demonstrated to show strong correlations with the degree of myelin content, therefore serving as an indicator of axonal degeneration [22]. Recently, accurate imaging of axonal degeneration *in vivo* has been established through optical coherence tomography (OCT), which measures the thickness of the RNFL [23]. It has been well established that Wallerian degeneration along retinal ganglion cell axonal fibers inevitably reaches the RNFL, which is unmyelinated and so axonal degeneration alone can be measured [24]. Given that optic neuritis is a prevalent initial clinical finding in MS, Klistorner et al., [24] have focused the clinical assessment of the disease by imaging the optic nerve. These investigators have shown a direct correlation between decreased amplitude and increased latency (markers of demyelination), measured with the newly developed mfVEP and the reduction in RNFL thickness (markers of axonal degeneration/loss). By performing these measurements on patients either suspected to have MS or those newly diagnosed post-acute optic neuritis, these investigators showed that axonal degeneration/loss was a prevalent finding in the context of neuroinflammation and demyelination [24]. A very recent study by these investigators has shown that the temporal RNFL is thinned in MS patients without previously presenting with optic neuritis and this was correlated with inflammatory lesions in the optic radiations, detected by DTI [25]. Such technology can provide high-resolution reconstruction of the retina (an anatomical site targeted during the early neurodegenerative process of MS). Therefore, OCT may be a plausible method to predict axonal degeneration and hence neurological impairment in MS patients with the added feature of studying the efficacy of neuroprotective therapies during the course of the disease. However, prospective multicenter studies have advocated for strict quality control criteria be implemented since boundary line errors due to poor scan quality and ring scan de-centration are common issues of clinical disagreement [26,27]. This has sparked the implementation of essential quality control criteria, identifiable as “OSCAR IB” (see [26]) and brought about debate of its clinical validity as an imaging biomarker purely on protocol and generation of artefacts. The clinical validity of OCT relies heavily on its inability to be the arbiter of CNS tissue injury and in particular since there still exist contradictory findings related to its ability in differentiating between the various subtypes of MS [27].

A means by which the degree of axonal degeneration may be studied involves the use of magnetic resonance spectroscopic (MRS) imaging. MRS allows for the detection of changes in metabolites such as N-acetyl aspartate (NAA), a marker of axonal integrity [28]. Reduced levels of NAA can be interpreted as potentiated axonal damage during the course of neurological diseases that involve axonopathy [28]. Whole brain MRS has successfully shown significant reduction in NAA and NAA/Creatine (NAA/Cr) ratio in CIS and RRMS compared with normal healthy controls [29-31]. However, these changes were not correlated with EDSS, limiting the clinical utility of these data. Reduction in NAA and NAA/Cr ratio was found in normal appearing white matter (NAWM) of RRMS and SPMS and interestingly, these changes were correlated with EDSS [32-39]. Importantly, it was found that the reduction in NAA levels demonstrated within NAWM of frontal and parietal brain areas were more evident in progressive manifestations of MS than RRMS [39]. Furthermore, studies have demonstrated that the reduction in NAA levels was more significant in T1 hypointense Gd-unenhancing lesions than acute lesions and no significant relationship between T2 hyperintense lesions with NAA levels [35-38]. These studies reflect the clinical relevance in measuring altered NAA levels during the course of MS as a biomarker for axonal damage in NAWM and both acute and chronic inflammatory lesions.

However, the MRS signal obtained for NAA may not necessarily correlate with tissue atrophy and axonal damage *per se*. In fact measurements obtained from the corpus callosum of patients with CDMS, via the sensitive diffusion fractional anisotropy, could demonstrate reduced size which correlated with patient EDSS but no such correlation could be seen with reduced NAA levels relative to tissue water [40]. Furthermore, while the MRS analysis from the spinal cords of patients primarily with SPMS clearly demonstrated reduced NAA levels with excellent correlation to only moderate EDSS and tissue atrophy [41]. Despite these encouraging findings, no statistically significant reduction in NAA levels could be attributed to NAWM areas in the frontoparietal cortices of these patients. It is also worth noting that although a 12-month longitudinal study was performed to evaluate the neuroprotective efficacy of interferon beta therapy, no restoration in NAA/Cr ratio could be observed in RRMS patients despite a reduction in CNS inflammatory lesions and relapse rates [42]. It is also not uncommon for NAA levels to be restored in RRMS in the lesion core and NAWM [43,44]. A further confounding factor to the limitation of NAA levels as a measure of axonal integrity is that fact that it is more likely a marker of neuro-axonal energy dysfunction, with an abrogated electron transport chain resulting in the plummeting levels of NAA [45]. Therefore, outside the added technical issues of accurate measurement [46], its pathobiological relevance as a predictive biomarker is still in question partly due to the heterogeneity of patient cohorts and the limited multicenter assessments performed.

However, these imaging techniques have also been used to study cortical adaptive functions in patients with RRMS, SPMS and PPMS [47,48]. In the initial disease presentation (RRMS), there seems no (or slight) reduction in NAA levels, however, a prominent reduction in NAA levels can be captured during progressive MS [47,49]. In accordance with progressive MS, changes in NAA levels within cortical grey matter were correlated with EDSS, auditive selective attention and cognitive performance [48,50,51]. Overall, changes in NAA in both NAWM and cortical grey matter can be strongly linked with physical disability. Indeed, a longitudinal therapeutic study of glatiramer acetate (FDA-approved disease modifying drug, Copaxone) treatment for RRMS, demonstrated partial recovery of NAA/Cr ratio [52]. Furthermore, an amalgamation of DTI and MRS using a 7 tesla magnet allowed for a more sensitive measurement of axonal damage within NAWM regions of the corpus callosum, demonstrating a clear reduction of NAA/Cr ratio, thereby supporting NAA reduction as a biomarker for to axonopathy during the course of MS [53].

As axonal degeneration can directly correlate with disease progression, a reliable prognostic biomarker for MS must target the detection of clear, reproducible axonal changes. Even though demyelination is a pathognomic feature of MS, a recent study has identified patients that exhibit a normal baseline MRI but present clinically with optic neuritis, have eventually manifested CDMS [54]. Demyelinating lesions, as detected through imaging techniques, cannot be a reliable diagnostic tool for MS. Although the levels of NF-H in the CSF, along with MRS measurement of NAA, are promising biomarkers of axonal damage during the clinical progression of MS, a combination of MRI (to detect demyelinating lesions) and MRS (detection of axonal changes) can be a powerful diagnostic and prognostic tool for nascent MS findings and progressive disease.

#### Molecular biomarkers: we are not there yet

**Figure 1:**
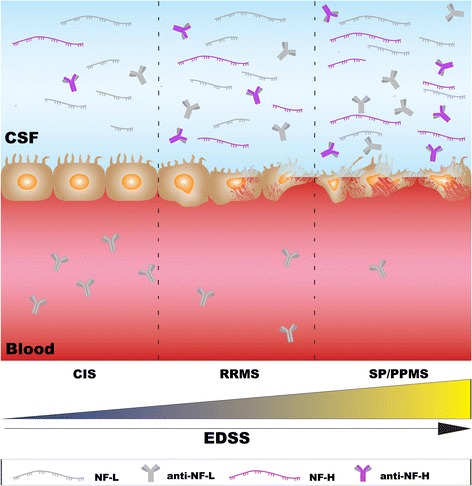
**Sequestered neurofilament and anti-neurofilament as potential prognostic biomarkers for progressive MS.** Both NF-L and NF-H can be released from CNS parenchymal cells into the CSF during clinical course of MS. The CSF levels of NF-L are consistently high throughout the disease progression, whereas the CSF levels of NF-H increase upon disease progression. Free NF-L and NF-H can be recognized by dendritic cells (antigen presenting cells) culminating in a further cycle of inflammation. These cells can therefore, activate B cells and T helper (T_H_) cells. Activated B cells can release autoantibodies against both NF-L and NF-H, which can potentiate axonal damage. The CSF levels of anti-NF-L and anti-NF-H increase during progressive MS, whereas the serum levels of anti-NF-L may decrease.

Detecting the presence of neuron-specific proteins in the CSF can be a powerful diagnostic/prognostic tool during MS, only if these proteins are shown to be directly correlative to the pathological sequelae of progression, beyond a causal association. Although lumbar puncture is considered as a safe method of obtaining CSF samples, its invasive nature, with requisite continuous sampling, is a limitation for the development of potential prognostic biomarkers of its incorporation in any clinical research study [55]. Despite this limitation, studies that have implemented CSF collection from patients during the progression of their MS have uncovered elevated levels of NF-L, a potential biomarker for axonal damage and a possible prognostic measure of progressive MS (Figure 1) [56]. The major drawback of these studies is the disparity in findings amongst specific patient groups with variable neurological presentations and tissue injury, bringing in to question the reproducibility of CSF NF-L levels as phenotypic biomarker.

As neurofilament is an integral protein that forms the axonal cytoskeleton, detection of neurofilament and its breakdown peptides within the CSF or serum can predict definitive axonal damage *in situ*. For instance, elevated levels of NF-L have been detected in CSF samples from individuals presenting with CIS which have subsequently progressed to CDMS [57]. More specifically in CIS, NF-L levels were increased when compared with control CSF samples obtained from patients with neuropsychiatric diseases of non-inflammatory etiology [58]. These elevated NF-L levels in the CSF were observed to be even greater in progressive MS or during relapse than those present in stable RRMS [59,60]. Circulating NF-L antigens in the CSF have also been postulated to trigger further autoimmunity against axons [61,62], experimentally illustrated through the immunization of mice with NF-L, which developed a progressive form of MS (predominant axonal damage with increased grey matter pathology) (Figure 1) [61,62].

The other major isoform, neurofilament heavy chain (NF-H), has also been found to be elevated in the CSF of all clinical MS types compared with normal healthy controls [63]. In particular, elevated levels have been reported in CIS with direct comparisons to neuropsychiartric diseases of non-inflammatory etiology [58], and these changes were correlated with declining EDSS [63]. These results emphasize that the level of NF-H in the CSF may relate to clinical disability. In comparison to NF-L, change in the CSF level of NF-H would appear to be more prominent in progressive MS [56]. However, again the major problem here is the confounding variable nature of the immunoassay results detecting NF-H levels observed from varying patient groups and different laboratories, rendering the data as possibly spurious [56].

It has recently been demonstrated that the immunotherapeutic, natalizumab, was shown to limit the rise in NF-L CSF levels during relapsing MS [64-66]. In addition, serum NF-H levels were shown to decrease during SPMS, when patients were treated with Na^+^ channel blocker, lamotrigine (based on serum lamotrigine adherence) [67]. The serum levels of NF-H were shown to correlate with clinical disability, EDSS and MRI cerebral atrophy [67] which further supports the notion that serum NF-H levels can manifest during disease progression and more likely in chronic MS (Figure 1). The possibilities of these clinically relevant studies are that both the NF-L and NF-H levels are stable molecules with excellent predictive nature of clinical outcome with regard to progression and quite possibly ongoing axonal damage following disease modifying therapeutic interventions. Despite this enthusiasm, MS patients on natalizumab still progress and the cerebral volume measurements in the lamotrigine trial did not advocate for neuroprotection, suggesting that we still await definitive evidence that NF-L and/or NF-H are clinically relevant biomarkers of axonal damage and predictive of MS progression.

### Mechanisms of axonal injury and degeneration during MS

#### Energy-dependent mechanisms of axonal degeneration

Following demyelination, the substantial energy demands placed upon axons, increase the stationary size of mitochondria and the speed of their transport along axonal microtubules [68]. Recent live *in vivo* imaging techniques applied in MOG_35–55_-EAE-induced mice revealed functional defects in intra-axonal mitochondria occurring even before prominent demyelination. This suggests that axonal mitochondria may be undergoing substantial damage prior to demyelination, indicating that an energy imbalance in axons may be the driver of the axo-glial degenerative phase. Recently, comprehensive reviews have covered the experiments that outline the unique mitochondrial deficits along with the generation of reactive oxygen species (ROS) and reactive nitrogen species (RNS) during the disease course of EAE leading to compromised axonal integrity [69,70] and so will not be discussed here.

Of particular importance to cortical atrophy attributed to MS pathology, whole-genome microarray analysis performed on post-mortem motor cortex tissue obtained from individuals who had exhibited SPMS, have demonstrated the down-regulation of neuronal-specific mitochondrial and *cox* genes, which encode for functional mitochondrial complex I and III activity [71]. In accordance with this finding, cortical chronic-active grey matter lesions also exhibited decreased complex IV activity along with multiple deletions of mitochondrial DNA [72]. On the other hand, in chronic-inactive lesions, increased complex IV activity was found [73], collectively indicating that there may be compensatory mechanisms during oxidative damage in cortical neurons [74]. This is in keeping with the increased mitochondrial density and complex IV activity observed in remyelinated axons from MS shadow plaques and in an ethidium bromide model of demyelination/remyelination [75] when compared with normally myelinated axons [75]. What this study highlights is that demyelination may cause delayed action potential propagation, possibly increasing axonal energy demand. However, when this energy demand exceeds axonal ATP production, it may undergo hypoxic-like axonal degeneration. Response to this state of hypoxia in axons may manifest as mitochondrial dysfunction due to an increase in NADPH oxidase or iNOS [2].

Dysregulation of mitochondrial transport can also severely impact the energy balance in axons, which may drive axonal degeneration. One scenario by which axonal degeneration may be initiated through mitochondrial dysfunction can be derived from the alterations exhibited in the histone deacetylases (HDACs). For the past decade, many groups have reported that HDAC inhibition was neuroprotective in MS (for review, see [76]). It was demonstrated that intraperitoneal administration of trichostatin A, a global inhibitor of HDACs, during MOG_35–55_-induced EAE could reduce disease severity. Furthermore, immunohistochemical analysis revealed that a higher axonal density in lumbo-sacral spinal cords could be demonstrated only in the trichostatin A treated group [77]. The molecular mechanism behind HDAC-mediated axonal degeneration was partially revealed by Kim et al., [78] who suggested that cuprizone-induced axonal damage can be triggered by Ca^2+^-dependent export of class I HDAC1 to the neuronal cytosol. This caused binding of HDAC1 with α-tubulin and kinesin motor proteins (KIF2A and KIF5), which were only detected in demyelinated areas, leading to an impairment of mitochondrial transport in neurons [78]. The same group also demonstrated a co-localization of cytosolic HDAC1 and SMI-32 in axons within demyelinated white matter, observed in human MS brain tissue [78]. As HDAC recruitment was found to be essential during remyelination upon lysolecithin-induced demyelination [79], the molecular mechanisms underlying HDAC inhibition mediated neuroprotection during neuroinflammation must be thoroughly characterized prior to clinical translation since they may target the myelin repair process.

Sirtuins (SIRT), class III family members of HDAC, have been raised as one of the candidate molecules that may serve as a therapeutic target to treat axonal degeneration in MS. Intravitreal administration of SIRT1 activator, SRT647 and SRT501, was shown to ameliorate retinal ganglion cell loss in PLP_139–151_-induced EAE within the SJL/J mouse model [80]. The same group also administered an oral dose of resveratrol, another SIRT1 activator and demonstrated neuroprotection in the same EAE model. Normal retrograde vesicular transport within retinal ganglion cell axons in mice treated with resveratrol was confirmed by fluorogold uptake assay and there was no modulation of neuroinflammatory or remyelination mechanisms upon resveratrol treatment [81]. Further investigation of the mechanism responsible for axonal integrity suggested that the neuroprotection exhibited by resveratrol in retinal ganglion cells during neuroinflammation may have been derived through the attenuation of oxidative stress by increasing the expression of the mitochondrial enzyme, succinate dehydrogenase and promoting deacetylation of the peroxisome proliferator activated receptor co-activator 1-α (PGC-1α) [82]. Overexpression of human SIRT1 in neurons of MOG_35–55_-induced EAE mice demonstrated neuroprotection with reduced inflammation and demyelination. Increase in brain-derived growth factor (BDNF) was seen in SIRT1 overexpressed neurons, which may indicate that SIRT1 overexpression can restore BDNF function, subsequently facilitating axonal protection [83]. Although SIRT1 activation or overexpression in neurons can be neuroprotective during neuroinflammation, possible effects of SIRT1 on other cell types during demyelination or neuroinflammation must be vigilantly investigated.

A plausible reason to investigate other neural lineages may be as a result of the recent study by Rafalski et al., [84] which implied that SIRT1 inactivation specifically in neural stem cells, shown using a *nestin*-Cre/loxP-*sirt1* transgenic model, can improve remyelination upon lysolecithin-induced experimental demyelination as well as during MOG_35–55_-induced EAE [84]. As plasma levels of SIRT1 were found to be increased in MS patients when compared with non-neurological disease controls, it is also postulated that soluble/circulating SIRT1 is an adaptive response during MS and it may play an important role in disease pathogenesis [85]. Therefore, a more comprehensive understanding of the molecular mechanism governing axonal preservation achieved through the activity of SIRT1 must be achieved to classify it as a neuroprotective agent of therapeutic potential.

#### Current ion channel theory in axonal damage

**Figure 2:**
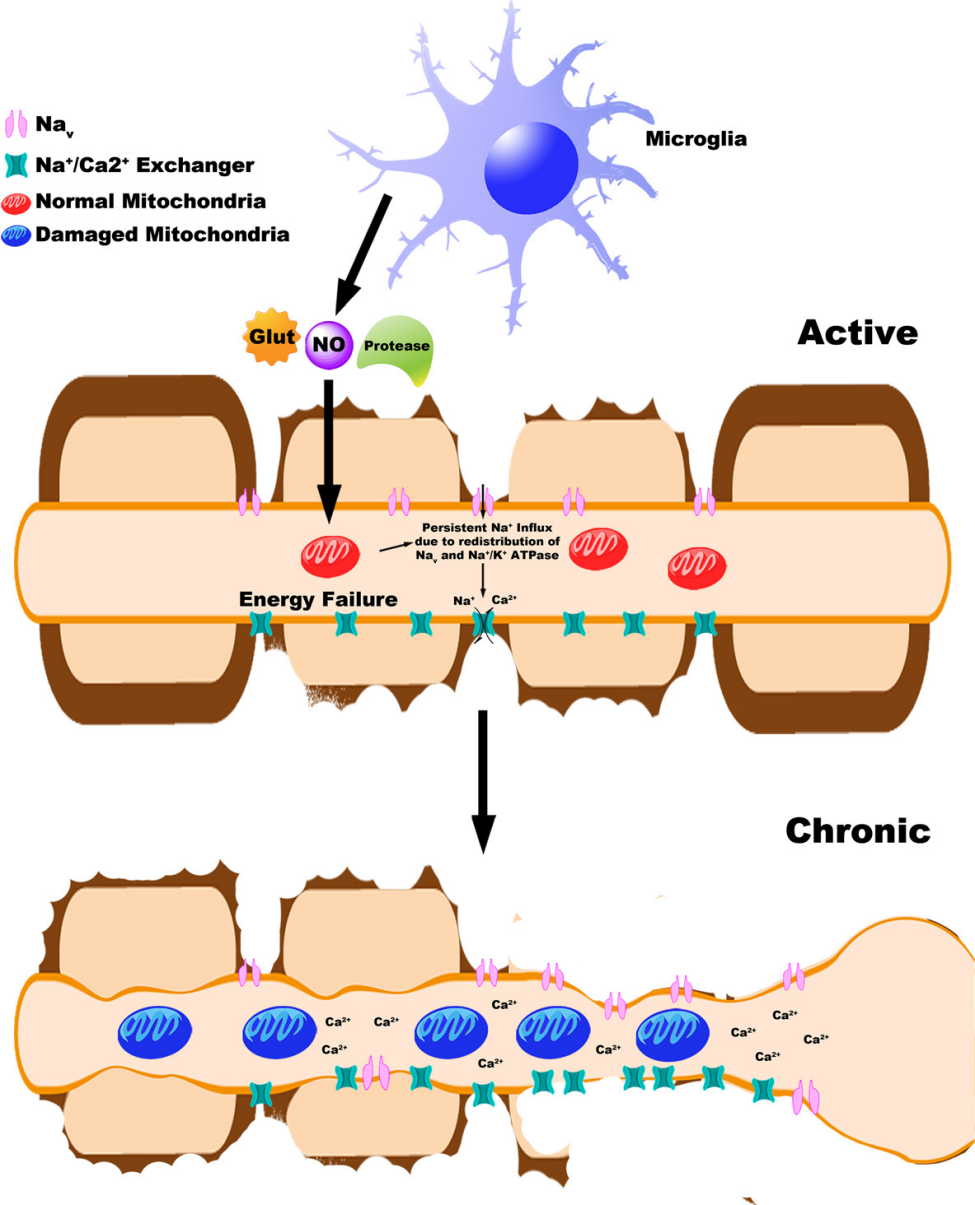
**Activated microglia and the redistribution of ion channels can potentiate axonal injury during MS.** Activated microglia can be a source of nitric oxide (NO), glutamate (Glut) or various proteases. Released free radicals, excitotoxic glutamate and various proteases can damage axonal mitochondria, leading to an imbalance in the energy demands/supply of axons. This along with the increased voltage-gated sodium channels (Na_v_) dispersed along denuded axons, can potentiate persistent Na^+^ influx. To compensate for this redistribution, increased expression of the Na^+^/Ca^2+^ exchanger functions in reverse, thereby cytotoxic levels of Ca^2+^ can mediate axonopathy.

The clinical use of sodium channel blockers to treat specific MS symptoms such as carbamazepine (the initial class of blockers) has fuelled interest in the mechanism by which persistent sodium influx can induce axonal injury during the symptomatic phase of MS. It has been posited that periods of remission from MS symptoms, may in-part, be related to a restoration of action potential conduction by Na^+^ channels along denuded axons [86]. Despite a lack of electrophysiological data to support this hypothesis, following demyelination, denuded axons can increase their density and number of Na^+^ channels in MS lesions [87]. Increased numbers of Na^+^ channels in demyelinated axons may produce an increase in axonal membrane potential oscillations [88], thereby potentiating neurotoxic levels of intra-axonal Ca^2+^, culminating in axonal degeneration [89]. ROS and RNS have been known to exert damage to axonal mitochondria with the consequential diminished energy supply to the axon [74]. This energy failure involves the accumulation of large quantities of Na^+^ within the axon due to the failed Na^+^ channels and persistent currents [90]. In response to this, the Na^+^-Ca^2+^ exchanger, which normally facilitates Na^+^ influx, instead functions in reverse mode to offset the rising levels of Na^+^ within the axon. However, the elevation of intracellular Ca^2+^ levels, a consequence of the failure in the ion exchanger, leads to the activation of a common pathway precipitating axonal degeneration [90] (Figure 2). Despite this tantalising hypothesis, no direct experimental evidence exists for this ion channel mechanism to be operative during the progression of MS symptoms.

Over the past few years, Stephen Waxman and colleagues have led the field in identifying how Na^+^ channels can initiate axonal degeneration in white matter tracts of the spinal cord of murine EAE models [91,92]. Notably, they have shown that the increased expression of Na_v_1.6 and Na^+^/Ca^2+^ exchanger is localized along damaged and demyelinated axons within many spinal cord tracts of EAE mice [89]. Mitochondrial dysfunction has been implicated in the reduction of axonal ATP levels, thereby rendering the Na^+^, K^+^ -ATPase defective, compromising the ability for the axon to set up appropriate Na^+^ transmembrane gradients and inevitably initiating the axonal degenerative process [93]. Blocking Na^+^ channels through the use of low dose TTX, phenytoin, lidocaine and flecainide, or blocking the Na^+^/Ca^2+^ exchanger through the use of bepridil has been successful in limiting the neurotoxicity after the induction of EAE [92,94-97]. These neuroprotective regimes tested in EAE suggest that one of the major mechanisms involved in axonal degeneration during neuroinflammatory diseases, is the persistent influx of Na^+^ into the axon ultimately leading to neurotoxic levels of Ca^2+^ (for review see [86]). Not surprisingly, the therapeutic rationale for the treatment of neurodegenerative mechanisms associated with the acute and chronic sequelae of MS is gaining momentum, particularly since the outcome is to limit disability. Limiting axonal degeneration has the added benefit of possibly prohibiting the spread of further oligodendrocyte and myelin damage proximal and distal to the initial axonal lesion due to the maintenance of reciprocal growth factor-dependent mechanisms, with the potential for profound neurological benefit [2]. These mechanistic dissections of Na^+^ channel blockers during neuroinflammatory disease have provided an evidence-based recruitment of patients in either PPMS, SPMS or RRMS for clinical phase trials in the US and the UK [98].

Another ion-channel blocking strategy set out to target Acid-sensing ion channel-1 (ASIC1), which is permeable to Na^+^ and Ca^2+^ and can contribute to the excessive accumulation of intracellular ions. Both genetic knockout and pharmacological blockade of this ion channel was shown to be neuroprotective in EAE-induced mouse spinal cord tissue [99]. As this ion channel was upregulated in MS lesions, in particular degenerative axons overexpressing βAPP [100], these studies have thus lead to the clinical translation of amiloride (blocker of ASIC1), with the PPMS treatment group achieving neuroprotection, confirmed by DTI and MRI [101]. This approach is promising and it is now currently in a phase II clinical trial for optic neuritis in the UK (ClinicalTrials.gov identifier: NCT01802489). However, the data is preliminary with a very small patient group analysed and loose criteria set as biomarkers for axonal damage such as axial diffusivity and altered rates in brain atrophy measured in treated patients over time. Furthermore, the data did not exclude a reduction in inflammation as the primary mechanism of neuroprotection again without achieving discrimination whether the amelioration of injurious intracellular axonal levels of Na^+^ and Ca^2+^ were the targets of amiloride treatment.

Although ion channel blockade holds substantial promise to limit axonal damage in MS, studies have reported significant worsening of disease upon withdrawal of specific ion channel blockers, a potentially fatal contraindication [96,98]. For example, it was demonstrated that administration of Na^+^ channel blockers during EAE significantly reduce clinical EAE scores, however, upon withdrawal of these drugs, marked increase in clinical severity were observed which were associated with a burst of inflammatory infiltrates within the CNS [96]. This may be a result of the drug modulating the Na^+^ channels immune cells, thereby exerting its neuroprotective effects via immunomodulation and not directly upon CNS axons as hypothesized. Furthermore, the Phase II clinical trial for lamotrigine (Na^+^ channel blocker), on secondary progressive MS patients showed exacerbation in motor function abnormalities, which were reverted upon a reduction in the lamotrigine dose [98]. These results clearly indicate the need for a sophisticated understanding of exact mechanism of action for such ion channel blockers. As other cell types do express ion channels, distinction of the modulatory effects of these partial blockers in immune cells and neurons would provide a more comprehensive understanding for their mechanisms of action. A very recent study by Al-Izki et al., [102] demonstrated a novel Na^+^ channel blocker (CFM6104) specifically targeted the early lesion (inflammatory penumbra) during EAE challenge in Biozzi ABH mice possibly through changes in p-glycoprotein [103], which was shown to be reduced both in EAE and MS lesions [102]. This novel drug reduced maximum severity of EAE disease score and improved motor-function during remission [102]. The authors have also found that there was no significant immunosuppression with this drug during EAE [102]. Although this study differentiated immunomodulation and neuroprotective drug actions, one should not overlook its possible rebound effects [96]. Furthermore, in the MOG_35-55_ EAE model a direct immunosuppressive role for the non-specific Na^+^ channel blocker Phenytoin highlighted caution for its use in Clinical Trials as the specific cellular target remains clearly unresolved [96]. Therefore, thorough basic science studies are required to clearly distinguish the mechanisms of drug action and to identify possible side/rebound effects before its clinical translation.

#### Cortical demyelination and atrophy – mechanisms governing progressive MS

Retrograde neurodegeneration in demyelinating grey matter lesions within the corpus callosum and hippocampus during neuroinflammation has recently been shown in a marmoset model of EAE as indicated by increased immunostaining of βAPP and decreased neuronal size and number [104]. Furthermore, increased SMI-34 (hyperphosphorylated NF-H) immunostaining was seen in demyelinating grey matter of the cerebral cortex of MS patients [105]. Attempts have been made to correlate these pathological changes with clinical disability (EDSS score), matching neuronal loss, axonal damage, and synaptic loss in demyelinating grey matter of hippocampus to memory loss [106-108]. Upon 12 weeks of cuprizone-mediated experimental demyelination, epileptiform spikes were measured by EEG/video monitoring [109]. Further investigations revealed extensive demyelination and Fluoro-Jade-C immunostaining in the hippocampus of these animals with long-term treatment of cuprizone [109]. MOG_35–55_-induced EAE within a cohort of *thy1*-YFP reporter mice revealed that significant reductions in inhibitory neurons along with pre-synaptic puncta observed within demyelinating grey matter throughout the hippocampus may be the reason for the reduced spatial learning exhibited by these mice [110]. These animal experiments have led to clinicopathological investigations of cortical demyelination and ensuing neurodegeneration in MS patients. Significant neuronal loss and reduction in size of neuronal somata were reported to present within the chronic demyelinated hippocampal grey matter of progressive MS brain tissue [106]. Moreover, a microarray-based gene expression study of demyelinated hippocampi revealed that there was a significant decrease in genes that are involved in axonal transport [108]. A profound deficit in anterograde axonal transport has been postulated from data generated in this study which demonstrated reduced mRNA levels of the kinesin gene family; KIF1A, KIF3A, KIF15, KIF5B, KIF5C and kinectin (KTN1) in demyelinating hippocampi of MS patients [108]. Decreased immunostaining of KIF1A was also documented for these lesions and may suggest a reduced learning enhancement/plasticity associated with hippocampal degeneration/synaptic integrity [111]. In addition, a potential impairment of retrograde transport was reflected by the alteration of mRNA levels of dynein molecules; DYNC1L12, DCTN1 and DNAH17 [108]. These results strongly advocate for a correlation between cortical demyelination and neurodegeneration, potentially representing memory deficits in MS patients.

Concerning the mechanisms of cortical demyelination in MS, the literature provides several likely hypotheses. Bruce Trapp’s group support the contention that alteration in glutamate uptake from hippocampal demyelination can cause neuronal energy imbalance thereby potentiating neurodegeneration [108]. A whole genome microarray approach revealed to these investigators that a decreased expression of glutamate receptors such as AMPA1, AMPA2 and AMPA3 in demyelinating hippocampi, may indicate an alteration of glutamate homeostasis [108]. Further bioinformatics analysis by the same group revealed that there were increased miR-124 levels, encoding for AMPA2 and AMPA3 in demyelinated hippocampi of MS patients. These changes could be reverted upon remyelination, hence, supporting the idea that myelin can influence glutamate homeostasis and demyelination can drive secondary neurodegeneration [112].

There exists evidence to suggest that the initiation of an autoimmune response can occur via the recognition of the glycoprotein autoantigen, contactin 2, on/near the endothelial cells of the grey matter by autoantibodies, type 1 helper T cells (Th1) and type 17 helper T cells (Th17) [113]. It is this process which enables the opening of the BBB, thereby allowing anti-myelin antibodies to gain access to the grey matter. Studies of PPMS have established a correlation between the degree of meningeal inflammation and cortical demyelination. This inflammatory state is believed to comprise increased concentrations of myelinotoxic and neurotoxic substances which in turn drive subpial pathology and demyelination, resulting in a greater disease severity [114]. Moreover, these investigators noted that monitoring brain atrophy over a 2-year period was an accurate prognostic indicator of disease progression. Further evidence of an immune response in the meninges as a pathophysiological mechanism of grey matter pathology is the presence of ectopic B cell follicle-like structures at these sites [115]. The extra-parenchymal structures are most commonly situated in the deep in-foldings of the cerebral sulci, and their numbers/extent are purported to be proportional to the size of cortical lesions present [116]. A large percentage of antigen experienced B cell clones present in the meningeal aggregates of 2/3 of MS brains have also been observed to be present in the corresponding parenchymal infiltrates [117]. The follicles are comprised of aggregates of B cells, immunoglobulins (IgA and IgG) and IgM-positive plasma cells. Identified by immunohistochemical staining for proliferating B cells, these follicles vary in size and distribution throughout the brain [115]. The study by Magliozzi and colleagues reveal the cytotoxic effects that these B cell aggregates have on cortical tissue. Essentially the follicles contain CD8-positive T-cells expressing interferon-gamma (IFN-γ) which cause cortical pathology either directly by cytotoxicity or indirectly by the induction of microglial cell activation [118]. Furthermore, it is evident that the CD8-positive cells in these follicles have the capacity to cause greater damage than those in the grey matter itself [118]. In a very recent development an animal model mimicking meningeal inflammation and cortical demyelination has been achieved through the stereotactic injection of tumor necrosis factor (TNF) and IFN-γ into the subarachnoid space following recombinant MOG immunization [119]. The data from this particular study implicates meningeal inflammation as a plausible initial pathology during neuroinflammation [119]. However, a relationship between meningeal inflammation and axonal pathology remains to be verified.

#### Integral axonal proteins: the role of microtubule transport in MS/EAE

##### Amyloid precursor protein (βAPP)

It is known that failure in axonal transport is one of the main causes of Wallerian degeneration [120]. Axonal transport deficits are often suggested to govern the pathologies which characterize classic neurodegenerative disorders, with Alzheimer’s disease as the archetypal pathology with dystrophic axons from such catastrophic dysfunction of the molecular transport machinery [121]. The dysregulation of axonal vesicular transport within long myelinated fibers of the CNS in MS patients is a candidate mechanism for the induction of axonal degeneration possibly sharing this feature with other neurodegenerative conditions. The theory gathers momentum when one investigates a classical histopathological marker of axonal damage, the accumulation of βAPP, which is normally not detectable by immunostaining as it is transported fast enough along axons if their integrity is maintained. Immunohistochemical staining of βAPP however is intense in swollen axons of chronic-active MS lesions and is now widely accepted as marker for impaired axonal transport to demonstrate axonal damage in MS [122].

##### Collapsin response mediator proteins (CRMPs)

**Figure 3:**
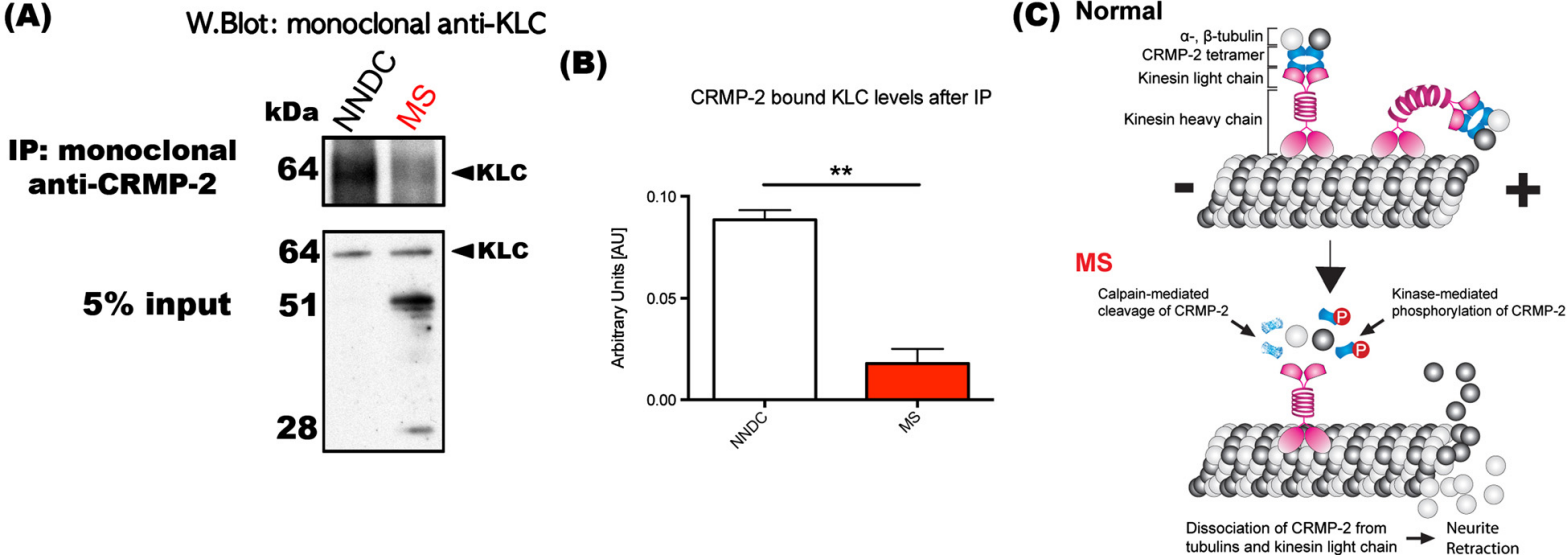
**Disturbed interaction between CRMP-2 and kinesin light chain in MS.** Tetrameric CRMP-2, may act as a cargo protein transporting molecules such as α- and β-tubulin heterodimers anterogradely with the aid of kinesin-1. CRMP-2 can be phosphorylated or cleaved during MS and this phosphorylation inhibits its binding to molecules impairing axonal transport. Inhibition of α- and β-tubulin heterodimers can promote the disassembly of polymerized microtubules. During MS, increased free calpain may potentiate cleavage of CRMP-2, impairing its function, leading to the failure of axonal transport and inevitably neurodegeneration. **(A)** (Top) Immunoprecipitation of CRMP-2, of brain white matter lysates from both non-neurological disease control (NNDC) and MS patients, followed by western immunoblot analysis using the monoclonal anti-kinesin light chain (KLC) antibody. (Bottom) Western immunoblot for KLC from the same brain samples loaded pre-immunoprecipitation (5% input of immunoprecipitation sample) using the monoclonal anti-KLC antibody. **(B)** Densitometric quantification (AU) of total KLC and KLC (after immunoprecipitation) from white matter lysates of NNDC and MS (*n* = 3; **P < 0.01, student’s *t*-test). **(C)** Concept diagram illustrating either cleavage or phosphorylation of CRMP-2 during MS culminating in the interference of the interaction between CRMP-2 with tubulin heterodimers and kinesin light chain, leading to microtubule disassembly.

CRMPs are a family of neuronal phosphoproteins [123] which regulate microtubule assembly as well as anterograde vesicular transport of important growth-related molecular cargo along neuronal microtubules [124,125]. CRMP-2 (the most well defined of the CRMPs) has already been shown to be phosphorylated by Cdk-5 at Ser522 [126,127], determined as the priming kinase, glycogen synthase kinase 3β (GSK-3β) at Thr514/509/Ser 518 [128,129] and also Rho kinase at the Thr555 position [130,131], all of which can mediate neurite retraction. Such phosphorylation disrupts the association of CRMP-2 with tubulin heterodimers so that tubulin is not able to be transported to the plus ends of microtubules for assembly, impeding directional growth of the neurite [125] (Figure 3). Importantly, phosphorylation of CRMP-2 reduces its binding to the kinesin-1 microtubule-related motor protein [132]. Since kinesin-1 is involved in anterograde vesicular axonal transport of molecules important for synaptic integrity and plasticity (e.g. BDNF receptor, TrkB) at the distal ends of axons [132], phosphorylation of CRMP-2 is expected to alter microtubule dynamics.

In terms of a pathogenic role attributed to the phosphorylated forms of CRMP-2, there is a clear link with the neurodegenerative processes of Alzheimer’s disease (Aβ-mediated phosphorylated CRMP-2) [133,134]. Recently, our lab showed that a Rho kinase II- specific phosphorylated form of CRMP-2 has profound importance in EAE disease progression, where substantial increase in the degeneration of axons within the spinal cord and optic nerve could be observed. We found that the pThr555CRMP-2 form demonstrated during the peak stage of EAE can be reduced through the administration of a function blocking antibody against Nogo-A (potent neurite outgrowth inhibitor) or alternatively, through the overexpression of the phosphomutant form of CRMP-2 by using an adeno-associated virus serotype 2 gene delivery system, could individually reduce the markers of degenerative axons appearing [135]. Our laboratory is now specifically targeting the phosphorylation of CRMP-2, which may be a plausible therapeutic regime in the treatment of progressive MS.

#### Calpains as de-stabilizers of the neuronal cytoskeleton in the MS model

Calpain-mediated cleavage of integral myelin proteins, including myelin basic protein, has been proposed as a prominent pathological mechanism leading to profound demyelination during EAE disease onset and progression [136]. Indeed, translational expression and activity of calpain have been reported to increase in inflammatory cells, activated microglia and astrocytes at the time of onset of clinical signs during EAE [137,138]. More importantly, targeted calpain inhibition in the nervous system has been shown to reduce inflammation and demyelination in the CNS as well as the clinical signs of EAE [139], suggested to be the result of attenuating the peripheral immune response to the CNS [140].

Calpains are members of the highly conserved calcium-dependent proteases capable of cleaving a vast array of cellular proteins [141]. Calpains are of specific interest in neurodegenerative disease since they can cleave cytoskeletal-related proteins such as the spectrin [142], tau [143], tubulin proteins and the CRMPs [144]. Since Ca^2+^-influx and the release of intracellular Ca^2+^ stores are plausible mechanisms by which axonal degeneration is potentiated during CNS injury and disease [141], the refractory transient increase in the deleterious calpains can potentially cause cleavage of cytoskeletal proteins and thus the collapse and degeneration of axons affected by these intracellular changes.

Two major isoforms of calpains exist namely, calpain-I (μ-calpain) activated at micromolar concentrations, and calpain-II (m-calpain) activated at up to millimolar concentrations [145]. In EAE, calpain is increased in axons due to the alteration of Na^+^ and subsequently Ca^2+^ influx. The sustained activation of calpain can lead to axonal degeneration, a hallmark of the pathophysiology of EAE [146]. Preservation of axonal integrity in EAE has been achieved by the administration of calpain-specific inhibitors such as CYLA [139]. This inhibitor can be synthesized by addition of cysteic acid (which can be actively transported into the brain through those mechanisms utilized by taurine to the leucyl-argininal of leupeptin), effectively crossing the blood–brain barrier [147]. Hassen and co-workers, [139], have recently shown that administration of intraperitoneal CYLA (2 mg daily) resulted in reduced levels of βAPP-positive and Na_v_1.6-positive axons (markers of axonal degeneration) in spinal cords of MOG_35–55_-induced EAE mice [139]. These data correlated with abrogated clinical scores in the CYLA-treated EAE mice and argue for the utilization of calpain inhibitors to limit axonal degeneration during neuroinflammatory CNS lesion formation, commonly characterized during MS pathogenesis.

#### Calpain and CRMP-2

There is now a more comprehensive argument for the provision of calpain inhibitors in MS and related neurological conditions. CRMP-2 has been identified as a common substrate for calpain-I during Wallerian degeneration *in vitro*, and calpain-mediated cleavage of CRMP-2 (Figure 3) may lead to its autophagic processing [148]. Calpain is reported to cleave CRMPs following ischemic brain injury in mice [149]. It cleaves CRMP-4 in primary rat cortical cultures as a result of either NMDA-induced excitotoxic insult or H_2_O_2_-induced oxidative stress [150]. Zhang and colleagues [151], have demonstrated calpain-mediated proteolysis of CRMP-1, −2 and −4 following neurotoxin treatment of primary cortical neurons and also following traumatic brain injury in the rat [151]. Hou and colleagues [152], have also found that calpain cleaves CRMP-3 following excitotoxic insult *in vitro* and cerebral ischemia *in vivo*. Interestingly, these investigators have shown that the cleaved product of CRMP-3, translocates to the nucleus and induces not only axonal retraction but also neuronal death [152]. This group has subsequently studied the expression patterns of all isoforms of CRMP (CRMP-1 to 5) and the proteolytic activity of calpain on CRMPs in ischemic brain injury, discovering that all CRMPs were highly expressed in apoptotic neurons [149]. Furthermore, synaptosomal CRMPs were found to be more susceptible to calpain cleavage than cytosolic CRMPs [149]. The physiological significance of this finding is unclear. However, it is possible that upon cleavage, synaptosomal CRMPs in particular, play a significant role in neuronal death with tantalizing implications to grey matter pathology in MS and clinical progression [153].

## Conclusion

Permanent neurological deficits in MS are governed by CNS axonal degeneration of major fiber tracts but the molecular mechanisms, which contribute to this damage, are poorly understood. The major contributors to axonal damage and loss may include: (1) axoplasm energy depletion caused by mitochondrial injury elicited through a hypoxic environment of sustained ROS and RNS, generated through activated microglia; (2) increased expression of Na_v_1.6 and the Na^+^/Ca^2+^ exchanger, which mediate cytotoxic levels of intra-axonal Ca^2+^ to compensate Na^+^ influx; (3) through the dysregulation of axonal transport machinery which may include abnormal modifications to the microtubule-associated proteins such as CRMP-2, thereby culminating in catastrophic damage of the axonal cytoskeleton. As current immunomodulatory therapies are limited in their ability to reduce relapses and in many ways ineffective during SPMS or PPMS, future therapies must be designed to halt the progression of clinical severity. From pre-clinical and clinical data, it is becoming evident that axonal injury is directly related to clinical progression. Therefore, ameliorating axonal damage during MS can limit the severity of disease to enhance the quality of life for MS sufferers. However, the mechanisms of potential therapy targeting axonal degeneration must be clearly defined with the potential effects upon non-neuronal cells, documented to ensure disease stage specificity without contraindications for MS patients.

## Abbreviations

MS: Multiple sclerosis
RNFL: Retinal nerve fiber layer
OCT: Optical coherence tomography
DTI: Diffusion tensor imaging
CNS: Central nervous system
NAA: N-acetyl aspartate
NF-L: Neurofilament light chain
NF-H: Neurofilament heavy chain
SPMS: Secondary progressive multiple sclerosis
PPMS: Primary progressive MS
CRMP-2: Collapsin mediator protein-2
RRMS: Relapsing-remiting multiple sclerosis
MRI: Magnetic resonance imaging
Gd: Gadolinium
EAE: Experimental autoimmune encephalomyelitis
mfVEP: Multifocal visual evoked potential
MRS: Magnetic resonance spectroscopy
NAA/Cr: NAA/Creatine
NAWM: Normal appearing white matter
ROS: Reactive oxygen species
RNS: Reactive nitrogen species
HDAC: Histone deacetylase
SIRT: Sirtuin
PGC-1α: Peroxisome proliferator activated receptor co-activator 1-α
BDNF: Brain-derived growth factor
ASIC1: Acid-sensing ion channel-1
Th1: Type 1 helper T cells
Th17: Type 17 helper T cells
IFN-γ: Interferon-gamma
TNF: Tumor necrosis factor
βAPP: Amyloid precursor protein
GSK-3β: glycogen synthase kinase 3β
